# Validity of routine reimaging of blunt renal trauma managed conservatively

**DOI:** 10.1097/MD.0000000000015135

**Published:** 2019-04-05

**Authors:** Ząbkowski Tomasz, Piasecki Piotr, Skiba Ryszard, Saracyn Marek

**Affiliations:** a Urology Department; b Department of Interventional Radiology; c Department of Endocrinology and Radioisotope Therapy, Military Institute of Medicine, Warsaw, Poland.

**Keywords:** blunt renal trauma, follow-up, kidney, reimaging

## Abstract

The purpose of this study was to determine the need of repeat follow-up computed tomography imaging in patients with renal trauma.

All patients who were admitted in the trauma center of the Military Institute of Medicine with a diagnosis of kidney injury from January 2008 to December 2017 were identified. A retrospective review of all patients’ medical records and radiologic imaging was conducted.

Data on the following factors were collected – patients’ demographics, mechanism of trauma, American Association for the Surgery of Trauma renal injury scale, injury severity score, laboratory examinations, multiorgan injuries, transfusion of fresh frozen plasma and packed red blood cells, time of surgical procedure in multiorgan injuries, length of hospital stay, and acute kidney injury.

This group consisted of 37 patients with left renal injuries, 32 with right renal injuries, and 5 with bilateral renal injuries. Renal trauma due to blunt injury secondary to a motor vehicle accident was noted in 45 patients, falling from a height in 14 patients, injury from battery in 4 patients, sports-related activities in 1 patient, and other factors in 10 patients.

Of the 63 patients treated conservatively due to multiorgan trauma or isolated trauma, values of morphology, serum creatinine and blood urea nitrogen, and ultrasonography in all patients did not reveal any pathological changes within earlier kidney damage.

The conservative treatment of grade I-IV renal injury in the American Association for the Surgery of Trauma scale provided good outcome and only involved noninvasive ultrasonography.

This study confirms that routine follow-up computed tomography imaging can be safely omitted in renal injuries graded I-IV providing that the patient remains in good clinical state.

## 1 Introduction

Renal trauma accounts for 1% to 5% of all traumas and is the most common genitourinary problem encountered by urologists in traumatic injury situations.^[1]^ Trauma is considered a global health problem, it is calculated more than 5 million deaths per year following to injury.^[2]^ In recent years, it created an impact toward conservative management, even in patients with severe trauma. A nonoperative approach to both blunt and penetrating renal injuries has contributed to higher rates of renal salvage and decreased morbidity compared with the primary operative management.^[3]^ To date, all reported series in adults used repeat computed tomography (CT) imaging to follow-up renal injuries. However, the pediatric literature has confirmed that ultrasonography (US) is a safe and effective alternative imaging modality used to monitor blunt renal trauma patients.^[4]^ The cost and radiation benefits of US are weighed against its lack of sensitivity and specificity as compared with CT.

The purpose of this study was to determine the need of repeat follow-up CT imaging in patients with renal trauma.

## 2 Methods

All patients who were admitted in the trauma center of the Military Institute of Medicine with a diagnosis of kidney injury from January 2008 to December 2017 were identified. A retrospective review of all patients’ medical records and radiologic imaging was conducted.

Data on the following factors were collected – patients’ demographics, mechanism of trauma, American Association for the Surgery of Trauma (AAST) renal injury scale, injury severity score (ISS), laboratory examinations, such as morphology, serum creatinine (sCr) concentration, and blood urea nitrogen (BUN) concentration, ethyl alcohol concentration, CT trauma scan, multiorgan injuries, transfusion of fresh frozen plasma (FFP) and packed red blood cells (PRBCs), time of surgical procedure in multiorgan injuries, length of hospital stay (days), and acute kidney injury (AKI). Patients with sCr level ≥1.5 mg/dL were diagnosed with AKI. Injury severity was defined using the ISS; data were obtained from the hospital trauma registry. The ISS correlates linearly with mortality, morbidity, and length of hospital stay.

**Figure 1:**
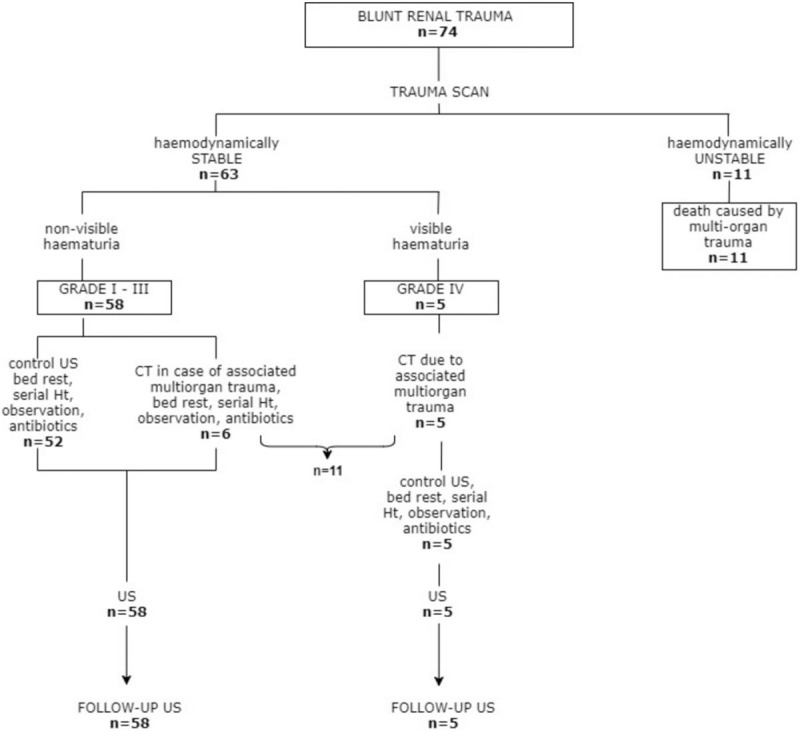
Blunt renal trauma management algorithm.

The modality and timing of follow-up renal imaging were dependent on the managing urologist but also related to multiorgan injuries. As a general rule, patients with isolated grade I renal injuries required no follow-up imaging. Patients with isolated renal injuries graded II-IV underwent follow-up US imaging. In 11 patients follow-up CT trauma scan was also performed in order to assess other, non-renal, injuries. No grade V renal injuries were included in this group (Fig. 1).

The statistical analysis was performed using STATISTICA software (StatSoft Inc, 2012). Parametric variables were reported as mean (± standard deviation [SD]). The Wilcoxon test was used for some nonparametric variables. An *α* risk less than 0.05 was considered significant.

## 3 Results

Of the 74 patients with renal trauma, 63 were included for follow-up imaging review. The remaining 11 patients were not available for follow-up due to death caused by multiorgan trauma.

**Figure 2:**
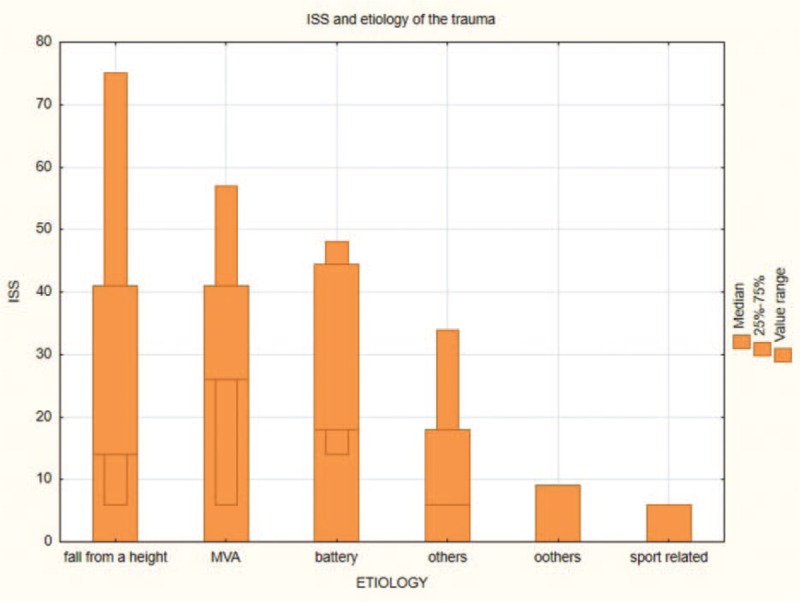
ISS and etiology of the trauma. ISS = injury severity score.

**Figure 3:**
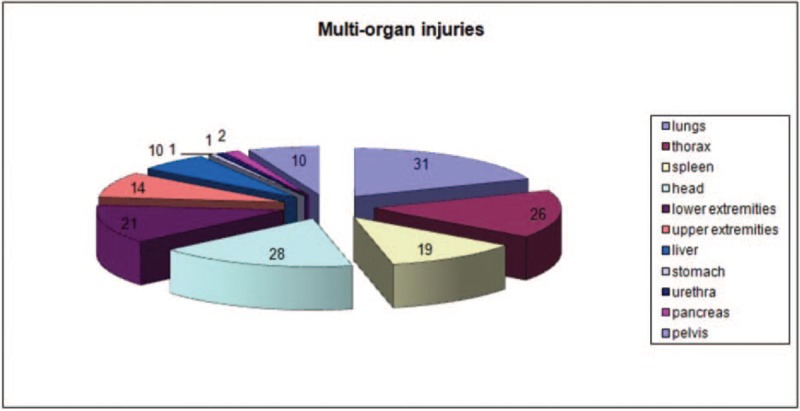
Multiorgan injuries.

**Figure 4:**
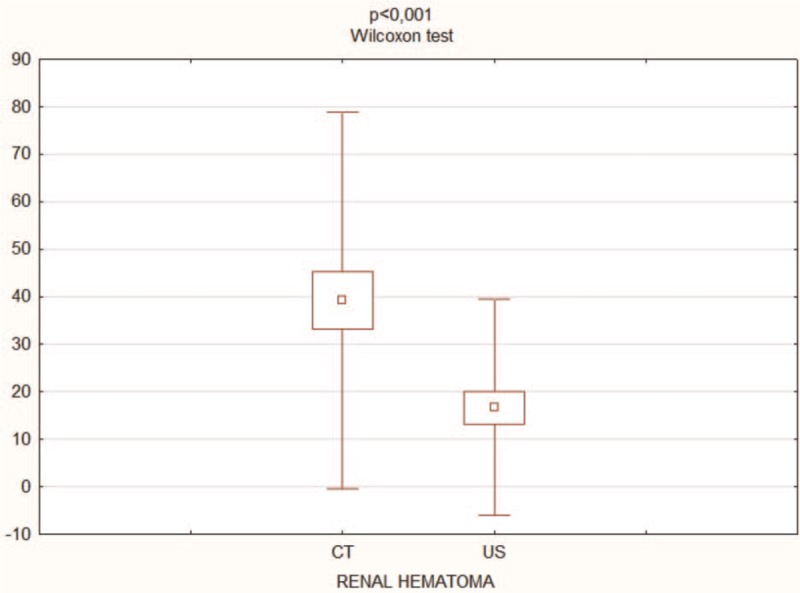
Comparison between size of renal hematoma in initial CT scans and follow-up US imaging. CT = computed tomography, US = ultrasonography.

**Figure 5:**
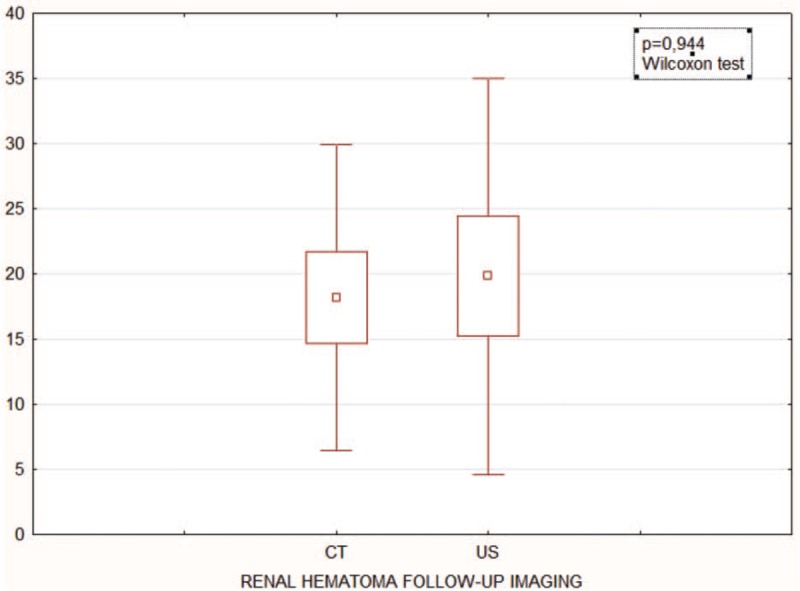
Comparison between size of renal hematoma in follow-up CT scans and follow-up US imaging. CT = computed tomography, US = ultrasonography.

The study group included 61 men (82.43%) and 13 women (17.57%) with an average age of 36 years (range: 17–76 years) who received a trial of conservative treatment of renal trauma and became the subjects of this report (Table 1 ). This group consisted of 37 patients with left renal injuries, 32 with right renal injuries, and 5 with bilateral renal injuries. Renal trauma due to blunt injury secondary to a motor vehicle accident was noted in 45 patients, falling from a height in 14 patients, injury from battery in 4 patients, sports-related activities in 1 patient, and other factors in 10 patients. According to the AAST scale, there were 69 cases of grade I-III renal trauma and 5 cases of grade IV renal trauma. The ISS ranged from 6 to 75 (median score = 32) (Fig. 2). Eighteen patients had isolated renal injuries while 56 patients had multiple organ injuries, such as lungs (n = 31), thorax (n = 26), spleen (n = 19), head (n = 28), lower extremities (n = 21), upper extremities (n = 14), liver (n = 10), stomach (n = 1), pelvis (n = 10), pancreas (n = 2), and urethra (n = 1) (Fig. 3). AKI occurred in 19 patients (25%) following posttraumatic shock. In initial CT scans mean renal hematoma diameter was 39.30 mm (SD-39.6 mm) and mean retroperitoneal hematoma was 49.2 mm (SD-39 mm). In follow-up, US imaging mean renal hematoma diameter was 16.69 mm (SD-22 mm) and mean retroperitoneal hematoma was 11.9 mm. Follow-up US imaging showed statistically significant decline in the size of the renal hematoma comparing to the initial CT scans (*P* < .001, Wilcoxon test) (Fig. 4). There were 11 out of 63 patients with follow-up CT imaging due to severe multiorgan injuries. Mean diameter of renal hematoma in the first CT was 55.63 mm (SD-42 mm) comparing to 18.18 mm (SD-11.7 mm) in the follow-up CT and 19.18 mm (SD-15 mm) in US imaging. In this group, 4 of 11 patients had retroperitoneal hematoma. Mean diameter in first CT was 64.75 mm (SD-43 mm) comparing to 20.5 mm (SD-24 mm) in follow-up CT and 20 mm (SD-23 mm) in US imaging. In each patient, both follow-up imaging methods gave comparable results, when it comes to the decrease in the size of the hematoma (*P* = .944, Wilcoxon test) (Fig. 5). Twenty patients (27%) were under the influence of alcohol; the median value of ethyl alcohol level was 2.09 per-mille. The average length of hospital stay of patients ranged from 1 to 129 days (mean, 15 days). The time of surgical procedure in multiorgan trauma ranged from 30 minutes to 12 hours and 15 minutes. Blood transfusion of 2 to 23 units of PRBC and 1 to 19 units of FFP was necessary for patients with multiorgan trauma.

**Table 1:**
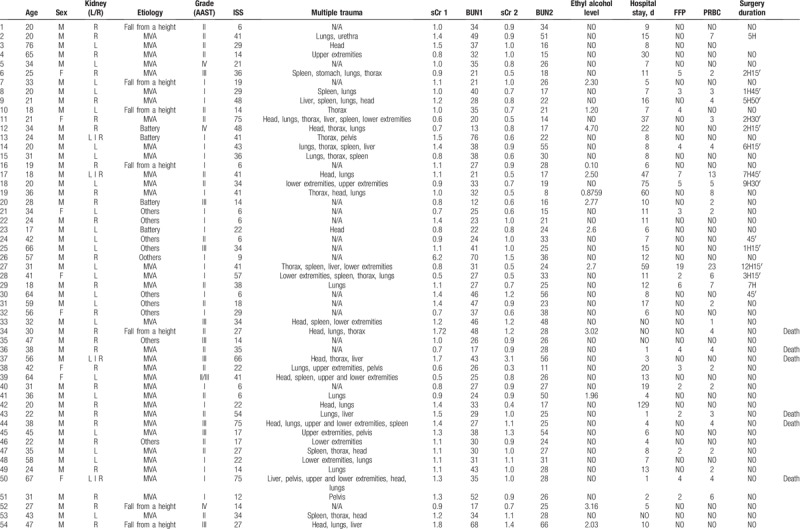
General characteristics of the cohort.

**Table 1 (Continued):**
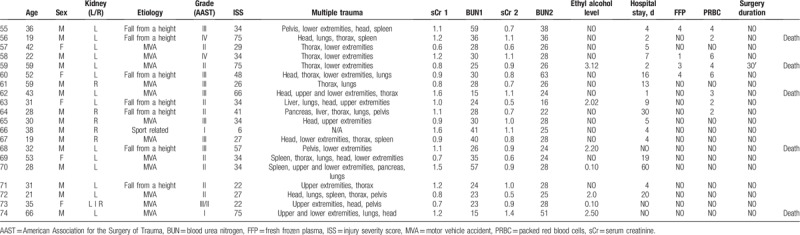
General characteristics of the cohort.

## 4 Follow-up

Of the 63 patients treated conservatively due to multiorgan trauma or isolated trauma, values of morphology, sCr and BUN, and US in all patients did not reveal any pathological changes within earlier kidney damage.

The surgical duration of other organs did not influence on renal function. In all patients with AKI after surgical procedures within multiorgan trauma and pharmacotherapy, normal renal function recovered with normalization of sCr and BUN.

**Figure 6:**
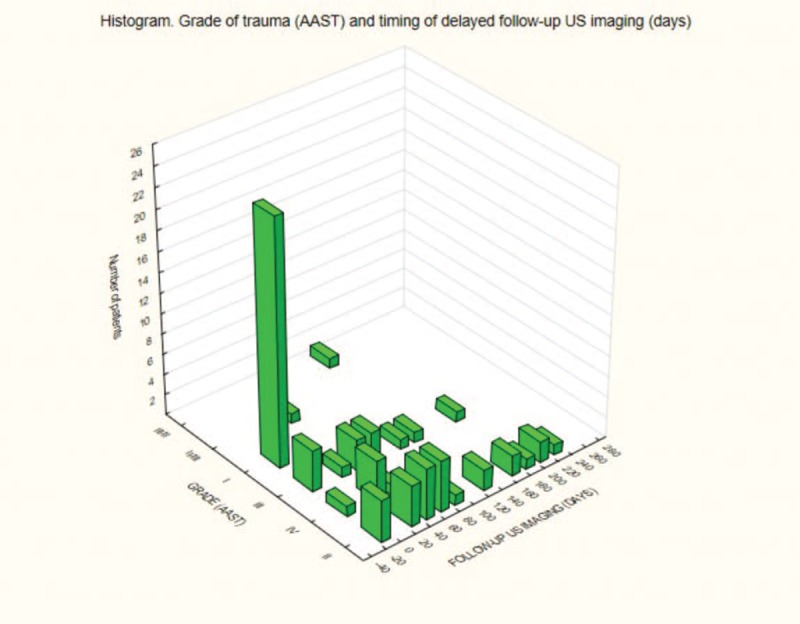
Grade of trauma (AAST) and timing of delayed follow-up US imaging (d). AAST = American Association for the Surgery of Trauma, US = ultrasonography.

The conservative treatment of grade I-IV renal injury in the AAST scale provided good outcome and only involved noninvasive US. The mean time from injury to delayed follow-up US imaging (days) was 47.3 days (range 30–240, SD = 62.15) (Fig. 6).

## 5 Discussion

The majority of renal injuries assessed at the trauma centers can be treated nonoperatively with a high success rate.^[5]^ CT is the most often used in the diagnosis of renal injury, which allows for rapid assessment and accurate injury grading according to the AAST-organ injury scale I.^[6]^ Imaging of renal trauma is well-established in the initial assessment of renal trauma, however, there is a lot of inconsistent reports on the effectiveness of follow-up imaging in these patients.^[7]^ In addition, there is no study examining the value of reimaging after penetrating renal injuries.

The rationale for repeat imaging is early identification and treatment of complications. Additional indications for repeat imaging aims to accept the potential evolution of an injury or to evidence a modality of the injured area of renal parenchyma.

Davis et al. confirmed that routine reimaging in patients with renal trauma after 48 hours without an indication was narrowly beneficial and contributed to change the treatment in less than 1%. This data present that reimaging can be safely omitted within grade I-IV injuries, providing that the patient is clinically well. However, in patients with grade V parenchymal injury, routine reimaging may be beneficial within early detection of complications, although the diagnostic yield is low.^[8]^

According to Bukur et al, selective follow-up imaging after renal injury supported by clinical and laboratory results seems to be safe and should be taken into consideration. In the case of patients with penetrating injuries who require surgical management, the lowest threshold for reimaging should be applied.^[3]^

Kieran et al. claimed that the lack of clinical or laboratory irregularities indicate that the benefits of routine follow-up imaging in renal trauma is low. A selective reimaging supported by clinical deterioration would have detected all complications in their study. Therefore, the authors recommend not to do routine reimaging in renal injuries graded I-III and in renal injuries graded IV without urinary extravasation. The development of grade IV injuries with urinary extravasation and grade V injuries should be controlled using a repeat US and CT imaging.^[9]^

The more selective approach contributes to the significant cost reduction and radiation exposure reduction, especially in case of long-term risks related to CT imaging and renal trauma in young patients.^[10,11,12]^

Mingoli et al confirmed that hemodynamically stable patients do not always require surgical exploration, because major renal trauma may be treated spontaneously or using minimally invasive procedures. In addition, the analysis of Mingoli et al showed a lower length of hospital stay of nonoperative management versus operative management of blunt renal trauma. The authors suggest that nonoperative management may be safely performed thereby operative management such as laparotomy, kidney resection, and nephrectomy may be avoided. These strategies contribute to hospital cost reduction.^[13]^ In the study of Matthews et al, spontaneous healing was observed in 87% of patients with renal injury and urinary extravasation.^[14]^ Furthermore, in the study of Haas et al, it was reported that the use of ureteral stents contributed to a high renal salvage rate in patients with renal trauma and urinary extravasation.^[15]^

Sujenthiran et al reported a comparative study of nonoperative versus operative management of renal trauma. Nonoperative management included ureteric stenting, percutaneous drainage in case of massive perirenal hematoma and also angioembolization in case of active bleeding from renal parenchyma. Moreover, it was confirmed that overall mortality, renal preservation, and length of hospital stay was significantly decreased in the nonoperative management group.^[16]^

In the study of Erlich et al, the majority of renal trauma is managed nonoperatively using careful monitoring, reimaging and minimally invasive procedures. These procedures included angioembolization in case of active bleeding and endourological stenting in case of urine extravasation.^[17]^

In this study, it was confirmed that AKI appears only after multiorgan trauma and represents 78.94% of multiorgan trauma and 25.67% of all trauma patients. In other words, none of patients with isolated trauma of any grade developed the AKI. Ten patients (13.51%) died, and 90% of deaths were noted in multiorgan trauma patients. 4/10 deaths occurred in a group of patients under alcohol influence. Therefore, we can deduce that alcohol acts a protective role.

The CT scan was rarely used as a follow-up diagnostic method because of the potential renal toxicity of contrast media.^[18]^ The regular reassessment was based on the US scan. However, in the 11 presented cases, there was an urgent need to perform the CT trauma scan as well in order to reassess multiorgan injuries. Nonetheless, the authors believe that the 2 imaging methods can be compared, mainly because collecting the sufficient data which would take many years. The outcome of such comparison shows that both methods present similar results.

## 6 Conclusion

Both CT and US scans gave comparable results of the size of renal hematoma.

The US imaging may be used as method of choice in follow-up evaluation in patients with renal injuries.

This study confirms that routine follow-up CT imaging can be safely omitted in kidney injuries graded I-IV providing that the patient remains in good clinical state. However, in case of patients with grade V parenchymal injury, routine CT reimaging may be beneficial within the detection of complications. These results confirmed that we can contribute to decreasing unnecessary radiation exposure and the cost of unhelpful investigations.

AKI after posttraumatic shock is transient and the kidneys return to their normal function. Patients with grade I trauma may be monitored by a family physician.

## Author contributions

TZ conceived the idea for the study. TZ and SM contributed to the design of the research. TZ and PP, SR were involved in data collection. TZ analyzed the data. All authors edited and approved the final version of the manuscript.

**Conceptualization:** Tomasz Ząbkowski, Saracyn Marek.

**Data curation:** Tomasz Ząbkowski, Skiba Ryszard.

**Formal analysis:** Tomasz Ząbkowski, Piasecki Piotr.

**Investigation:** Tomasz Ząbkowski, Piasecki Piotr.

**Methodology:** Tomasz Ząbkowski.

**Resources:** Skiba Ryszard.

**Supervision:** Tomasz Ząbkowski, Piasecki Piotr, Skiba Ryszard.

**Visualization:** Tomasz Ząbkowski, Skiba Ryszard, Saracyn Marek.

**Writing – original draft:** Tomasz Ząbkowski, Saracyn Marek.

**Writing – review and editing:** Tomasz Ząbkowski, Saracyn Marek.
